# Supplementary material for: The Gene paaZ of the Phenylacetic Acid (PAA) Catabolic Pathway Branching Point and ech outside the PAA Catabolon Gene Cluster Are Synergistically Involved in the Biosynthesis of the Iron Scavenger 7-Hydroxytropolone in Pseudomonas donghuensis HYS
Source: Int J Mol Sci. 2023 Aug 10;24(16):12632. doi: 10.3390/ijms241612632 (PMC10454607; doi:10.3390/ijms241612632)
Supplement: Supplementary file 1 [file ijms-24-12632-s001.zip › ijms-2489490-supplementary.pdf]

Title

The gene *paaZ* of the phenylacetic acid (PAA) catabolic pathway branching point and *ech* outside the PAA catabolon gene cluster are synergistically involved in the biosynthesis of the iron scavenger 7-Hydroxytropolone in *Pseudomonas donghuensis* HYS.

Panning Wang, Yaqian Xiao, Donghao Gao, Yan Long * and Zhixiong Xie *

Hubei Key Laboratory of Cell Homeostasis, College of Life Sciences, Wuhan University, Wuhan 430072, China;
2015202040019@whu.edu.cn (P.W.); 2018102040012@whu.edu.cn (Y.X.); 2021202040036@whu.edu.cn (D.G.);
* Correspondence: skyjck4@whu.edu.cn (Y.L.); zxxie@whu.edu.cn (Z.X.)

Supplemental Materials

Screening and characterization of transposon mutants with reduced siderophore production.

As identified in our previous work [20, 22], to define the essential genes involved in the biosynthesis of siderophores, random transposon insertion mutation was carried out using the vector pBT20 and modified chromeazurol S (CAS) agar assays in *P. donghuensis* HYS. The mutants deficient in siderophore yields were obtained in *P. donghuensis* HYS. Then, sequence alignment of the transposon-flanking sequences of the insertion sites in these 9 mutants with the genome sequence of *P. donghuensis* HYS indicated that the mutant genes were mainly located on cluster 2 (Table S1 and Figure 1A).

These 9 mutant strains were inserted in 5 different genes in cluster 2 (Table S1 and Figure. 1A). Six of these mutants (M7, M54, M1, M37, M49, M50) showed insertions in five ORFs (*orf17*, *orf19–orf21*) may arrange sequentially in the genome, and the other three mutants (M31, M40, M60) inserted in the *orf26*. The siderophores production of the 9 mutant strains was significantly lower than that of the wild-type HYS, suggesting that the genes *orf17*, *orf19–orf21,* and *orf26* corresponding to the 9 mutant strains were related to the biosynthesis of siderophores in *P. donghuensis* HYS.

Table S1. Characteristics of the 9 transposon mutants of *P. donghuensis* HYS.

Mutant[a]	Insertion site[b]	Insertion gene function[c]	Relative siderophore production[d]	
			CAS plate	Liquid medium
M7	Scaffold18 84439-84440	Phenylacetic acid degradation protein	0.18 ± 0.01	0.17 ± 0.01
M54	Scaffold18 84549-84550	Phenylacetic acid degradation protein	0.15 ± 0.01	0.16 ± 0.00
M1	Scaffold18 86359-86360	Phenylacetic acid degradation protein	0.14 ± 0.00	0.16 ± 0.00
M37	Scaffold18 86766-86767	Phenylacetate-CoA oxygenase	0.18 ± 0.01	0.15 ± 0.00
M49	Scaffold18 87193-87194	Phenylacetate-CoA oxygenase subunit PaaA	0.12 ± 0.00	0.16 ± 0.00
M50	Scaffold18 88042-88043	Phenylacetate-CoA oxygenase subunit PaaA	0.15 ± 0.02	0.16 ± 0.01
M60	Scaffold18 92847-92848	Enoyl-CoA hydratase	0.20 ± 0.00	0.15 ± 0.00
M40	Scaffold18 93134-93135	Enoyl-CoA hydratase	0.16 ± 0.01	0.15 ± 0.00
M31	Scaffold18 93315-93316	Enoyl-CoA hydratase	0.15 ± 0.02	0.16 ± 0.00

[a]The mutant strains are displayed according to their relative positions on the scaffold of the HYS genome.
[b]The insertion site is located between the two bases shown.
[c]The possible functions of genes are based on genomic annotation of *P. donghuensis* HYS.
[d]The relative ferrider-producing capacity was the ratio of ferrider-producing capacity of mutant strain to ferrider-producing capacity of wild-type HYS. On the CAS test plate, the siderophore production capacity was the radius value of the chelated halos；In MKB liquid medium, it is the number of siderophore units (%) produced by bacteria per OD_{600} (see Materials and methods for details). Each value is the mean ± standard deviation of at least three independent experiments.

Table S2. Characteristics of genes in cluster 2 in *P. donghuensis* HYS.

Gene	No.of amino acid residues	Proposed Function(s)[a]	Organism	% identity[b]	% coverage	Significance (E value)	Accession no.
orf13	689	Phenylacetic acid degradation bifunctional protein PaaZ	*E.coli* K-12	57.8	98	0.0	THI66261.1
orf14	413	OprD family outer membrane porin	*E.coli* M34	80.87	100	0.0	MRF40643.1
orf15	513	Cation/acetate symporter ActP	*E.coli* K-12	69.6	100	0.0	HBP1329774.1
orf16	102	DUF485 family inner membrane protein	*E.coli* K-12	42.1	92	1e-21	HBP1325669.1
orf17	358	Phenylacetate-CoA oxygenase/reductase subunit PaaE	*E.coli* K-12	50.0	99	3e-119	THH46419.1
orf18	177	Phenylacetate-CoA oxygenase/reductase subunit PaaD	*E.coli* K-12	53.3	86	4e-53	THI66257.1
orf19	251	Phenylacetate-CoA oxygenase/reductase subunit PaaC	*E.coli* K-12	51.0	98	4e-87	THI66258.1
orf20	93	Phenylacetate-CoA oxygenase/reductase subunit PaaB	*E.coli* K-12	58.9	96	1e-38	NP_415907.1
orf21	329	Phenylacetate-CoA oxygenase/reductase subunit PaaA	*E.coli* K-12	68.0	93	2e-164	NP_415906.1
orf22	436	Phenylacetate—CoA ligase PaaK	*E.coli* K-12	68.2	97	0.0	THI66250.1
orf23	403	3-oxoadipyl-CoA thiolase PaaJ	*E.coli* K-12	70.3	99	0.0	NP_415915.1
orf24	142	Hydroxyphenylacetyl-CoA thioesterase PaaI	*E.coli* K-12	44.4	95	1e-37	THI66252.1
orf25	505	3-hydroxyacyl-CoA dehydrogenase PaaH	*E.coli* K-12	47.1	96	1e-134	NP_415913.1
orf26	263	2-(1,2-epoxy-1,2-dihydrophenyl)acetyl-CoA isomerase	*E.coli* K-12	62.8	99	4e-109	THH46417.1
orf27	256	2,3-dehydroadipyl-CoA hydratase	*E.coli* K-12	57.9	98	2e-96	NP_415911.1
orf28	198	Phenylacetic acid degradation protein PaaY	*E.coli* K-12	58.5	97	5e-80	THI66248.1
orf29	307	Phenylacetic acid degradation operon negative regulatory protein PaaX	*E.coli* K-12	43.6	99	2e-87	THH46412.1

[a]CoA, coenzyme A.

[b]Similarity values are for the most similar proteins, determined by BLASTP analysis, whose functions and source organisms are also shown.

Analysis and identification of transcriptional units and promoters of cluster 2.

In order to further determine the relationship between the genes *orf17*, *orf19–orf21*, *orf26* screened by transposon insertion mutation in cluster 2 and the biosynthesis of siderophore, we analyzed and identified the transcriptional units of these genes. *orf17–orf21* are arranged in a 5 kb region of the HYS genome, which have the same transcription direction as the downstream genes *orf14–orf16* and the upstream genes *orf22-orf27* (Figure 1A). In addition, the distance between adjacent gene sequences was close or partially overlapped, so we speculated that there may be co-transcriptional relationships between them.

Firstly, to search for and analyze potential promoters in this region, we use a promoter prediction program (http://www.fruitfly.org/seq_tools/promoter.html), and found possible promoters in front of *orf21* and *orf27* respectively (Minimum promoter score=0.8) (data not shown).

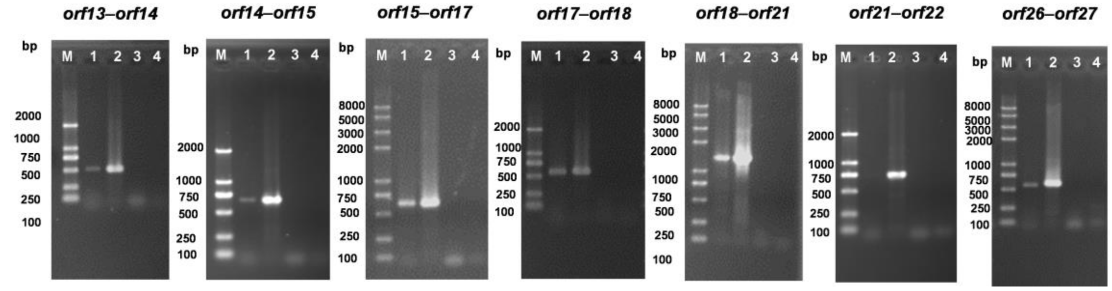

Figure S1. RT-PCR of RNA extracted from *P. donghuensis* HYS with ORF connecting primer pairs (*orf13–orf14* RT-1 plus *orf13–orf14* RT-2, *orf14–orf15* RT-1 plus *orf14–orf15* RT-2, *orf15–orf17* RT-1 plus *orf15–orf17* RT-2, *orf17–orf18* RT-1 plus *orf17–orf18* RT-2, *orf18–orf21* RT-1 plus *orf18–orf21* RT-2, *orf21–orf22* RT-1 plus *orf21–orf22* RT-2, *orf26–orf27* RT-1 plus *orf26–orf27* RT-2). The different lanes represent different templates: lanes M, DNA Maker; lane 1, cDNA; lane 2, genomic DNA as a positive control; lane 3, RNA without reverse transcription as a negative control; lane 4, ddH_2O as a blank control.

Furthermore, to confirm the transcriptional relationships among these genes, primer pairs (Table.S5) connecting neighboring (intergenic region) ORFs were used to do the Reverse transcription (RT-PCR).The results showed that products generated using HYS cDNA as the template were similar to those obtained using genomic DNA as the template for these (*orf13–orf14*, *orf14–orf15*, *orf15–orf17*, *orf17–orf18*, *orf18–orf21, orf21–orf22, orf26–orf27*) connecting primer pairs, in addition to the *orf21–22* connecting primer pairs without target products formed using HYS cDNA as the template (Figure S1). The negative and blank control using genome-removed RNA and ddH_2O as the template respectively did not produce the target bands. The target fragments amplified by genomic cDNA as template were extracted and sequenced, and the sequencing results were all correct, which were the corresponding cross-gene target sequences. The above Reverse transcription PCRs results indicated that there was a co-transcriptional relationships of *orf13–orf21*, and the cotranscription of *orf26–orf27*, but

no co-transcriptional relationships between *orf21* and *orf22*. According to the promoter prediction and co-transcriptional analysis results, the predicted promoter in front of *orf21* was used for further studies on *orf17–orf21* and the predicted promoter upstream of *orf27* was used for further studies on *orf26.*

Detection of the phenylacetic acid metabolic ability of related genes on gene cluster 2 by using phenylacetic acid as a single carbon source.

The arrangement and annotation of cluster 2 were shown in Figure.1A. Through NCBI BLASTP analysis, proteins encoded by genes in cluster 2 shared high similarity (43–70%) with proteins encoded by genes in PAA cluster genes of *E.coli* K-12 (Table S2), suggesting that cluster 2 may be a phenylacetic acid metabolic cluster.

To investigate whether 7-HT related genes in cluster 2 were involved in phenylacetic acid metabolism, phenylacetic acid was added to the M9 minimal medium as a sole carbon source. In the experiment, it was found that HYS, Δ*orf13* (Δ*paaZ*), Δ*orf17* (Δ*paaE*), Δ*orf18* (Δ*paaD*), Δ*orf19* (Δ*paaC*), Δ*orf20* (Δ*paaB*), Δ*orf21* (Δ*paaA*), Δ*orf26* (Δ*paaG*), Δ*orf17–21* (Δ*paaEDCBA*) and Δ*orf26* (Δ*paaG*) strains were cultured in M9 medium with 0.4% glucose (Glu) as a single carbon source to the stationary phase, compared with the wild-type HYS, each knockout strain had no obvious growth difference, indicating that the knockout of these genes did not affect the glucose utilization of *P. donghuensis* HYS (Figure S2A). When phenylacetic acid was used as the sole carbon source for cultivation to the stationary phase, compared with the wild-type strain HYS, the knockout strains Δ*orf13*(Δ*paaZ*), Δ*orf17* (Δ*paaE*), Δ*orf18* (Δ*paaD*), Δ*orf19* (Δ*paaC*), Δ*orf20* (Δ*paaB*), Δ*orf21* (Δ*paaA*), Δ*orf26* (Δ*paaG*), Δ*orf17–21* (Δ*paaEDCBA*) could not grow (FigureS 2B), indicating that these deletion strains cannot utilize phenylacetic acid. The knockout strains Δ*orf17* (Δ*paaE*), Δ*orf18* (Δ*paaD*), Δ*orf19* (Δ*paaC*), Δ*orf20* (Δ*paaB*), Δ*orf21* (Δ*paaA*), Δ*orf26* (Δ*paaG*), Δ*orf17–21* (Δ*paaEDCBA*) exhibited significant growth deficiency when phenylacetic acid was used as sole carbon source (Figure S2 B).

In summary, the sole carbon utilization experiments showed that the knockout of *orf13*, *orf17–21,* and *orf26* genes in gene cluster 2 caused the strain unable to use phenylacetic acid for growth, so it was preliminarily clarified that these genes in cluster 2 were involved in the catabolism of phenylacetic acid in *P. donghuensis* HYS. Moreover, the phenylacetic acid degradation pathway is involved in the biosynthesis of 7-HT in *P. donghuensis* HYS (Figure 1), which provides a direction for the study of the 7-HT biosynthesis pathway.

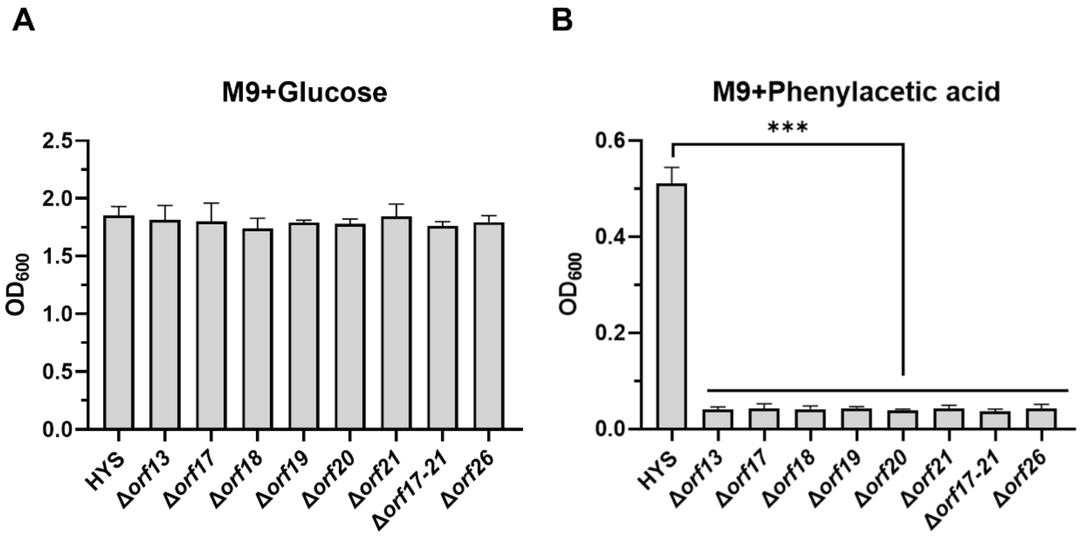

Figure S2. Growth of wild-type HYS and each knockout strain in a single carbon source at the stationary phase. (A) M9 minimal medium with 0.4% glucose as a single carbon source. (B) M9 minimal medium with 0.6 mg/ml phenylacetic acid as a single carbon source. Each value is the average from three different cultures ± the standard deviation. OD_{600}, optical density at 600 nm. *** $p < 0.001$, Student's *t*-test.

Table S3. Characteristics of Paaz-ECH homologous protein[a] in *P. donghuensis* HYS

Description	Scientific Name	Max Score	Total Score	Query Cover	E value	Per. Ident	Acc. Len	Accession[b]
(R)-hydratase [(R)-specific enoyl-CoA hydratase]	*P. donghuensis* HYS	63.5	63.5	90%	1×10^{-13}	32.45%	156	UW3_RS0113785
NodN (nodulation factor N)	*P. donghuensis* HYS	54.7	54.7	50%	2×10^{-10}	32.18	151	UW3_RS0112810

[a]Similarity values are for the most similar protein, determined by BLASTP analysis.
[b]Gene id in *P. donghuensis* HYS.

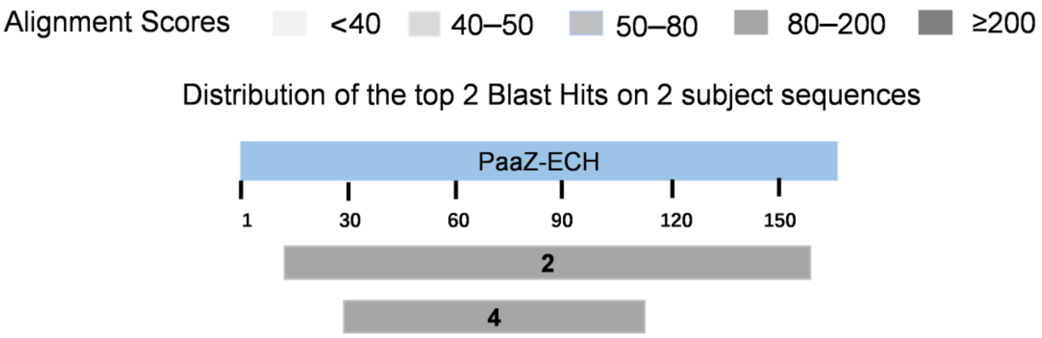

Figure S3. Distribution of the 2 proteins (ECH and NodN) with homology to the C-terminal ECH domain of ORF13 (PaaZ) obtained by whole genome alignment search of *P. donghuensis* HYS.

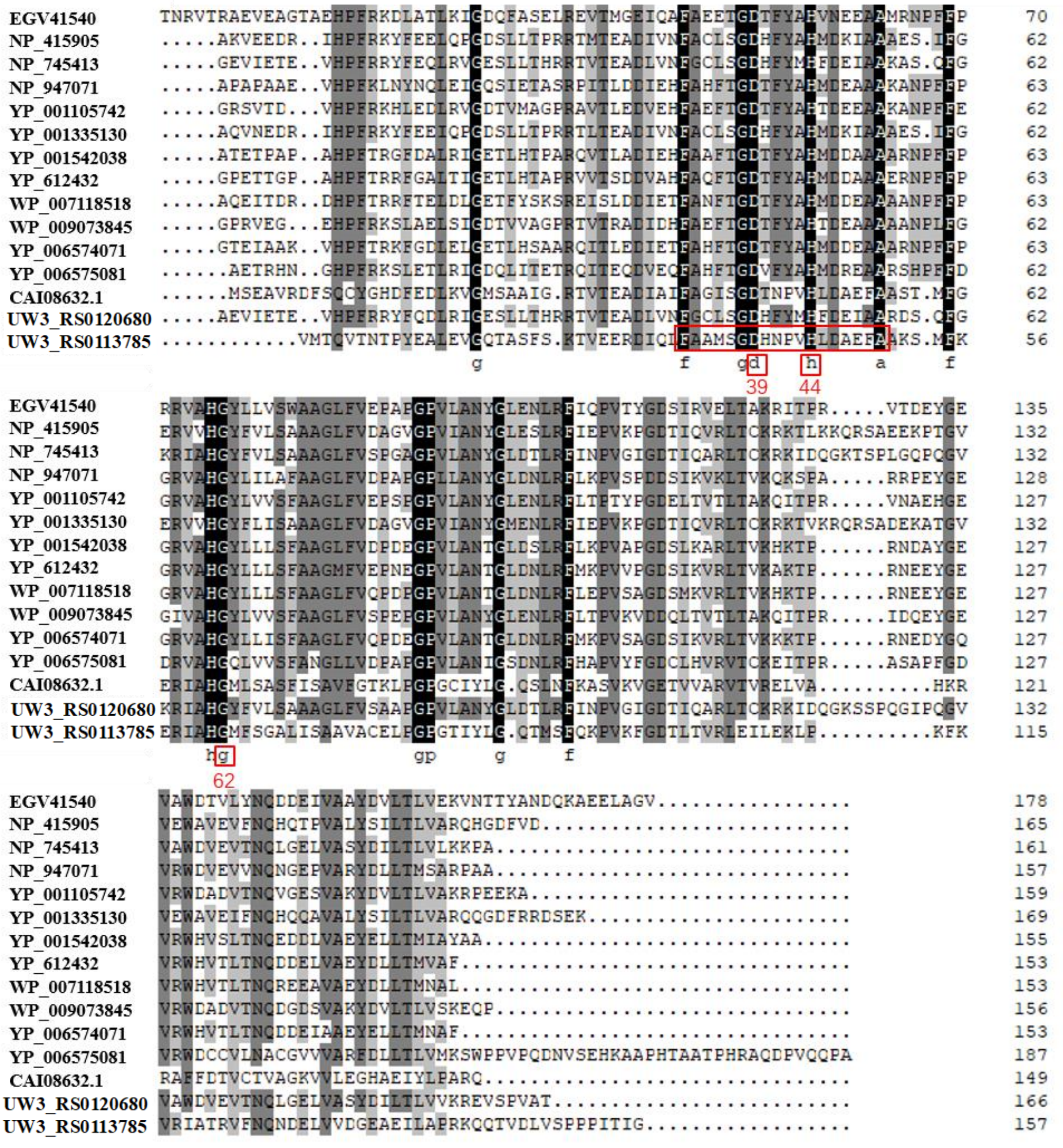

Figure S4 Partial sequence alignment of PaaZ-ECH domain of a selection PaaZ orthologues with ECH and highlight the selected residues and conserved consensus sequence. Protein sequences for alignment from a selection of bacteria (EGV41540:*Corynebacterium glutamicum*, NP_415905: *Escherichia coli*,NP_745413:*Pseudomonas putida*, NP_947071:*Rhodopseudomonas palustris*, YP_001105742:*Saccharopolyspora erythraea*, YP_001335130: *Klebsiella pneumoniae*, YP_001542038: *Dinoroseobacter shibae*, YP_612432: *Ruegeria sp.*, WP_007118518: *Oceanibulbus indolifex*, WP_009073845: *Streptomyces sp.*, YP_006574071: *Phaeobacter inhibens* PaaZ1, YP_006575081: *Phaeobacter inhibens* PaaZ2, CAI08632.1: *Aromatoleum aromaticum* ECH-Aa, UW3_RS0120680: *P. donghuensis* HYS PaaZ, UW3_RS0113785: *P. donghuensis* HYS *ech*. Inside the red rectangle is the ECH-like conserved consensus sequence of ECH of *P. donghuensis* HYS.The red squares indicate the assumed key conserved residues (D-39,H-44,G-62) of the ECH domin in *P. donghuensis* HYS, each residue was mutated into Alanine by site mutation. Consistent residues (D,H,G) of *E. coli* PaaZ-ECH have been identified. The number indicates the position of the residue in the sequence of the ECH domain.

Table S4 Bacterial strains and plasmids used in this study

Strain or plasmid	Description[a]	Source or reference(s)
E. coli S17-1 λpir	*thi pro hsdR recA*; chromosomal RP4–2; (Tc::Mu) (Km::Tn7) Tp^r Sp^r	[47]
Pseudomonas strains		
HYS	Wild-type *P. donghuensis* HYS^T; Cm^r	[18,19,20]
Δ*orf17*	*paaE* deletion strain	This study
Δ*orf18*	*paaD* deletion strain	This study
Δ*orf19*	*paaC* deletion strain	This study
Δ*orf20*	*paaB* deletion strain	This study
Δ*orf21*	*paaA* deletion strain	This study
Δ*orf17–21*	*paaEDCBA* deletion strain	This study
Δ*orf26*	*paaG* deletion strain	This study
Δ*orf13* (*paaZ*)	*paaZ* deletion strain	This study
Δ*ech*	*ech* deletion strain	This study
Δ*NodN*	*NodN* deletion	This study

Δ*paaZ*Δ*ech*	*paaZ*, *ech* deletion strain	This study
Δ*ech*/pBBR2	Δ*ech* complemented with pBBR1MCS-2	This study
Δ*paaZ*Δ*ech*/pBBR2	Δ*paaZ*Δ*ech* complemented with pBBR1MCS-2	This study
Δ*ech*/pBBR2-*ech*	Δ*ech* complemented with pBBR2-*ech*	This study
Δ*paaZ*Δ*ech*/pBBR2-*ech*	Δ*paaZ*Δ*ech* complemented with pBBR2-*ech*	This study
Δ*ech*/pBBR2-*ech*D39A	Δ*ech* complemented with pBBR2-*ech*D39A	This study
Δ*ech*/pBBR2-*ech*H44A	Δ*ech* complemented with pBBR2-*ech*H44A	This study
Δ*ech*/pBBR2-*ech*G62A	Δ*ech* complemented with pBBR2-*ech*G62A	This study
Δ*ech*/pBBR2-*paaZ*	Δ*ech* complemented with pBBR2-*paaZ*	This study
Δ*ech*/pBBR2-*paaZ*E258Q	Δ*ech* complemented with pBBR2-*paaZ*E258Q	This study
HYS/pBBR2	HYS complemented with pBBR1MCS-2	This study
HYS/pBBR2-*paaZ*	HYS complemented with pBBR2-*paaZ*	This study
HYS/pBBR2-*paaZ*E258Q	HYS complemented with pBBR2-*paaZ*E258Q	This study
**Plasmids**		
pEX18Gm	Gene replacement vector with MCS from pUC18; Gm^r *oriT sacB*	[48]
pEX18Gm-*orf17*-UD	Gene replacement vector for *orf17*	This study
pEX18Gm-*orf18*-UD	Gene replacement vector for *orf18*	This study
pEX18Gm-*orf19*-UD	Gene replacement vector for *orf19*	This study
pEX18Gm-*orf20*-UD	Gene replacement vector for *orf20*	This study
pEX18Gm-*orf21*-UD	Gene replacement vector for *orf21*	This study
pEX18Gm-*orf17–21*-UD	Gene replacement vector for *orf17–21*	This study
pEX18Gm-*orf26*-UD	Gene replacement vector for *orf26*	This study
pEX18Gm-*orf13*(*paaZ*)-UD	Gene replacement vector for *orf13*(*paaZ*)	This study
pEX18Gm-*ech*-UD	Gene replacement vector for *ech*	This study
pEX18Gm-*NodN*-UD	Gene replacement vector for *NodN*	This study
pBBR1MCS-2	Mobilizable broad-host-range cloning vector; Km^r	[49]
pBBR2-*paaZ*	*EcoRI-SalI* fragment containing the *paaZ* gene of HYS cloned into pBBR1MCS-2	This study
pBBR2-*paaZ*E258Q	The *EcoRI-SalI* fragment containing the *paaZ* gene with E258Q site mutation cloned into pBBR1-MCS2	This study
pBBR2-*ech*	*XhoI-HindIII* fragment containing the *ech* gene of HYS cloned into pBBR1MCS-2	This study
pBBR2-*ech*D39A	The *XhoI-HindIII* fragment containing the *ech* gene with D39A site mutations cloned into PBBR1-MCS2	This study
pBBR2-*ech*H44A	The *XhoI-HindIII* fragment containing the *ech* gene with H44A site mutations cloned into PBBR1-MCS2	This study
pBBR2-*ech*G62A	The *XhoI-HindIII* fragment containing the *ech* gene with G62A site mutations cloned into PBBR1-MCS2	This study

[a]Cm, chloramphenicol; Gm, gentamicin; Km, kanamycin; Sp, spectinomycin; Tp, trimethoprim; MCS, multiple-cloning site.

Table S5. Oligonucleotide primers used in this study

Primer	Sequence (5’-3’)[a,b]	Description
***orf13*-up-1**	GGAATTCAACCGCAACTTCAACGCCAGCTT	Construction of Δ*orf13*
***orf13*-up-2**	GCTCTAGACATCGGGTGTCCTCCGGTTTC	Construction of Δ*orf13*
***orf13*-down-1**	GCTCTAGATGATCTTGAACTGCGCCCCAC	Construction of Δ*orf13*
***orf13*-down-2**	CAAGCTTACTGCTCAACGCCGTGTCC	Construction of Δ*orf13*
***orf13*-M-1**	TTGTCGCAGCGCACCCAT	Verification of Δ*orf13*
***orf13*-M-2**	TGCCTTCAACCCACAACTGA	Verification of Δ*orf13*
***orf17*-up-1**	GGAATTCGTGGTCAGCGTGGTGGAGCTTGGGA	Construction of Δ*orf17*
***orf17*-up-2**	CGAGCTCCATGGTCGTTCTCCATCAGGCGCAG	Construction of Δ*orf17*
***orf17*-down-1**	CGAGCTCTAAGCCCAAGGAGCAGAAGACGA	Construction of Δ*orf17*
***orf17*-down-2**	CAAGCTTTCGCCGAGATGCCGAGGAAG	Construction of Δ*orf17*
***orf17*-M-1**	CACGTCGGCGAAGGTGTATTTAC	Verification of Δ*orf17*
***orf17*-M-2**	TTCCTGCCACGAGCCTACCC	Verification of Δ*orf17*
***orf18*-up-1**	GGAATTCCGATTTCGCCGTGACCAT	Construction of Δ*orf18*
***orf18*-up-2**	CGAGCTCGGCAATCAGTTCACCAGG	Construction of Δ*orf18*
***orf18*-down-1**	CGAGCTCTGAGCGCACATTGCTGCGCCT	Construction of Δ*orf18*
***orf18*-down-2**	CAAGCTTGCGGTTGCCGTAGAGCAGGGTGA	Construction of Δ*orf18*

***orf18*-M-1**	CATCGATACGACCGTTGTACAGA	Verification of Δ*orf18*
***orf18*-M-2**	GCAACTGGCTGGACTACGC	Verification of Δ*orf18*
***orf19*-up-1**	GGAATTCTGGGACGAGTTCTACGAAGTGC	Construction of Δ*orf19*
***orf19*-up-2**	CGAGCTCCAGGTAGGGGATCAGGTC	Construction of Δ*orf19*
***orf19*-down-1**	CGAGCTCCCCGATGCAACCTGGTGAACTGATT	Construction of Δ*orf19*
***orf19*-down-2**	CAAGCTTACTGGCTGAGCAATTCGGTATGGC	Construction of Δ*orf19*
***orf19*-M-1**	CGACACCGCATCACGGGTTTCAT	Verification of Δ*orf19*
***orf19*-M-2**	CGCTTCATCGACCAAACCATC	Verification of Δ*orf19*
***orf20*-up-1**	GGAATTCGCCAGGGCTACGAAATGCTCC	Construction of Δ*orf20*
***orf20*-up-2**	CGGATCCCATGGCGGGCTCTCCGGTCA	Construction of Δ*orf20*
***orf20*-down-1**	CGGATCCGGGCATATGTAACATGACCCAG	Construction of Δ*orf20*
***orf20*-down-2**	CAAGCTTAGCGGCGCAAATGGTAGGTGA	Construction of Δ*orf20*
***orf20*-M-1**	GGCGGCTGTCCAGGTAGAA	Verification of Δ*orf20*
***orf20*-M-2**	ACAACGGCAAGGCCAAGTA	Verification of Δ*orf20*
***orf21*-up-1**	GGAATTCGGTCGAGGAACAGCTGCTTAA	Construction of Δ*orf21*
***orf21*-up-2**	CGAGCTCCAGTTGTGCGTACATGGTG	Construction of Δ*orf21*
***orf21*-down-1**	CGAGCTCTGACCGGAGAGCCCGCCATGT	Construction of Δ*orf21*
***orf21*-down-2**	CAAGCTTCAGCAGGTTGCGGTAGTCG	Construction of Δ*orf21*
***orf21*-M-1**	ATGGTAGGTGACCTCTTTCAACGC	Verification of Δ*orf21*
***orf21*-M-2**	GCCCTGCCGATGATCCGCTAT	Verification of Δ*orf21*

***orf17–21*-up-1**	G<u>GAATTC</u>TGATCCGCTATCGCACCC	Construction of Δ*orf17–21*
***orf17–21*-up-2**	C<u>GAGCTC</u>CAGTTGTGCGTACATGGT	Construction of Δ*orf17–2*1
**o*rf17–21*-down-1**	C<u>GAGCTC</u>TAAGCCCAAGGAGCAGAAGACGA	Construction of Δ*orf17–21*
***orf17–21*-down-2**	C<u>AAGCTT</u>TCGCCGAGATGCCGAGGAAG	Construction of Δ*orf17–21*
***orf17–21*-M-1**	CGGCGAAGGTGTATTTAC	Verification of Δ*orf17–21*
***orf17–21*-M-2**	GTCTGGGAGGACCATTTCT	Verification of Δ*orf17–21*
***orf26*-up-1**	G<u>GAATTC</u>GCTGCCGTCAATGGCTACGC	Construction of Δ*orf26*
***orf26*-up-2**	C<u>GGATCC</u>CATGGTATCTGCCTTTGGTTGC	Construction of Δ*orf26*
***orf26*-down-1**	C<u>GGATCC</u>TGAGCATGACTGCCCTCAGTC	Construction of Δ*orf26*
***orf26*-down-2**	C<u>TCTAGA</u>GGCGGTGTCCTTGATGCACTCGG	Construction of Δ*orf26*
***orf26*-M-1**	CAGCATTGGCGCCGTGGT	Verification of Δ*orf26*
***orf26*-M-2**	GCGTCGAATACCGAACAGGTCAC	Verification of Δ*orf26*
**RT-*rpoB*-1**	CGGGAGCGACCAAAGATCAG	Real-time qPCR
**RT-rpoB-2**	CGTACTCCAGGGCAGCATTG	Real-time qPCR
**RT-*orf13*-1**	TGAGCGAACGCAAGGAGCA	Real-time qPCR
**RT-*orf13*-2**	TATGGCTGCCGACGAACGA	Real-time qPCR
**RT-*orf17*-1**	GAACAACTCGAAATGAATCCGT	Real-time qPCR
**RT-*orf17*-2**	AAGACCTGAAGAACCGCTACC	Real-time qPCR
**RT-*orf18*-1**	TGGCGTGACAAAGCCTGCGTCCTCC	Real-time qPCR
**RT-*orf18*-2**	GCGTGGTGGAGCTTGGGATCGTTCG	Real-time qPCR

**RT-*orf19*-1**	CCCGAGCGGCGCAAATGG	Real-time qPCR
**RT-*orf19*-2**	CGCAACTGGCTGGACTACG	Real-time qPCR
**RT-*orf20*-1**	CAGCGGCTCTTTCTCGTC	Real-time qPCR
**RT-*orf20*-2**	ACCCTGTTTGAAGTATTCGTGC	Real-time qPCR
**RT-*orf21*-1**	GGTCAGGAGCATTTCGTAGCCC	Real-time qPCR
**RT-*orf21*-2**	GCACAACGGCAAGGCCAAGTAC	Real-time qPCR
**RT-*orf26*-1**	GTTGACGGCACAGATGACC	Real-time qPCR
**RT-*orf26*-2**	CTGTTGCTGACCGCCGAAG	Real-time qPCR
***orf13–orf14*-RT-1**	CGCTCCTCGTGGCTGTA	Reverse transcription PCR
***orf13–orf14*-RT-2**	CGCCTTTCCGTTTCTCAA	Reverse transcription PCR
***orf14–orf15*-RT-1**	CCAGTTTAGCCGTGAAGC	Reverse transcription PCR
***orf14–orf15*-RT-2**	CCTTGCCACCTTAGGGATT	Reverse transcription PCR
***orf15–orf17*-RT-1**	CGAGATGCCGAGGAAG	Reverse transcription PCR
***orf15–orf17*-RT-2**	CGTGCCAGAGCTATCCA	Reverse transcription PCR
***orf17–orf18*-RT-1**	CATCGTTGGCAAAGGC	Reverse transcription PCR
***orf17–orf18*-RT-2**	GGAGGACGCAGGCTTT	Reverse transcription PCR
***orf18–orf21*-RT-1**	AAGCCTGCGTCCTCCA	Reverse transcription PCR
***orf18–orf21*-RT-2**	CTTCATCGACCAAACCATC	Reverse transcription PCR
***orf21–orf22*-RT-1**	CGGGCATCCAGTTCTT	Reverse transcription PCR
***orf21–orf22*-RT-2**	ATCCGCTATCGCACCC	Reverse transcription PCR

***orf26–orf27* RT-1**	CAGCGGGTTGTAGTAGCG	Reverse transcription PCR
***orf26–orf27* RT-2**	GCCTGGTCAGCGAAATCAC	Reverse transcription PCR
orf13-1	GCGTCGACAAACCGGAGGACACCC	*orf13* amplicon
orf13-2	CGGAATTCTCAAGATCAGGTTGCCACG	*orf13* amplicon
**(*orf13*)*paaZ*-E258Q-1**	AGCGAGTCGGC**TTG**GGCGCTGAAGG	*paaZ*E258Q
**(*orf13*)*paaZ*- E258Q-2**	CCCTTCAGCGCC**CAA**GCCGACTCGC	*paaZ*E258Q
***ech*-up-1**	CGGAATTCTGTCGTTCGGCTCGTT	Construction of Δ*ech*
***ech*-up-2**	GGCTGACACCTGGGTCATCACTTGTTTTTCCT	Construction of Δ*ech*
***ech*-down-1**	CCCAGGTGTCAGCCCGCCACCCAT	Construction of Δ*ech*
***ech*-down-2**	AGGACCGCAGCCTGTGGAACCG	Construction of Δ*ech*
***ech*-up-F**	CGGAATTCTGTCGTTCGGCTCGTT	Construction of Δ*ech*
***ech*-down-R**	CCAAGCTTACCGCAGCCTGTGGAACCG	Construction of Δ*ech*
***ech*-M-1**	TCGGCCACAGCATGGGCAGCTATAT	Verification of Δ*ech*
***ech*-M-2**	CGCCATGACTTGAGCGATGAAGCCA	Verification of Δ*ech*
***NodN*-up-1**	AGCGACATACATAGGGCATTGTGACTCTC	Construction of Δ*NodN*
***NodN* -up-2**	CTCCTGGTTGTCTTGACGGG	Construction of Δ*NodN*
***NodN*-down-1**	CCGTTGTACTGGTCGTCCTTGA	Construction of Δ*NodN*
***NodN*-down-2**	CTATGTATGTCGCTCTGCTTCGTCTGA	Construction of Δ*NodN*
***NodN*-up-R**	CGGAATTCTCCTGGTTGTCTTGACGGG	Construction of Δ*NodN*
***NodN*-down-F**	CCAAGCTTCCGTTGTACTGGTCGT	Construction of Δ*NodN*

***NodN*-M-1**	TTCCAGGCGATTGTCATGAAAAGCG	Verification of Δ*NodN*
***NodN*-M-2**	CACCAGTTGATAGGCACCCAGGGTA	Verification of Δ*NodN*
**RT-*ech*-1**	CCGAGTTCGCCGCCAAGAG	Real-time qPCR
**RT-*ech* -2**	GCTCGTCGTTCTGATTGAACAC	Real-time qPCR
ech-1	CGGAATTCAGAAGGAAAAACAAGTGATGACCCA	*ech* amplicon
ech-2	CCAAGCTTTCAGCCGATGGTGATGGGTGGCGGG	*ech* amplicon
***ech*-D39A—1**	ATCCAGGTGCACCGGGTTGTG**GGC**A	*ech*D39A
***ech*-D39A—2**	T**GCC**CACAACCCGGTGCACCTGGAT	*ech*D39A
***ech*-H44A—1**	ATCCAG**GGC**CACCGGGTTGTGGTCA	*ech*H44A
***ech*-H44A—2**	TGACCACAACCCGGTG**GCC**CTGGAT	*ech*H44A
***ech*-G62A—1**	AACAT**GGC**GTGGGCGATACGCTCCT	*ech*G62A
***ech*-G62A—2**	AGGAGCGTATCGCCCAC**GCC**ATGTT	*ech*G62A

[a]Underline sequences are restriction enzyme sites.

[b] Bold sequences refers to bases of the mutant amino acid site.
